# High inflammatory cytokines gene expression can be detected in workers with prolonged exposure to silver and silica nanoparticles in industries

**DOI:** 10.1038/s41598-024-56027-z

**Published:** 2024-03-07

**Authors:** Vahid Babaei, Azadeh Ashtarinezhad, Maryam Torshabi, Shahram Teimourian, Morteza Shahmirzaie, Jamileh Abolghasemi, Hamidreza Zeraatgar Gohardani, Eisa Kaveh Vernousfaderani, Farshad H. Shirazi

**Affiliations:** 1 Pharmaceutical Sciences Research Center, Shahid Beheshti University of Medical Sciences, Tehran, Iran; 2 Department of Occupational Health Engineering, School of Public Health, Iran University of Medical Sciences, Tehran, Iran; 3 Department of Dental Biomaterials, School of Dentistry, Shahid Beheshti University of Medical Sciences, Tehran, Iran; 4 Department of Medical Genetics, School of Medicine, Iran University of Medical Sciences, Tehran, Iran; 5 Department of Biostatistics, School of Public Health, Iran University of Medical Sciences, Tehran, Iran; 6 Department of Medical Laboratory Sciences, Tehran University of Medical Sciences, Tehran, Iran; 7 Department of Pharmacology and Toxicology, School of Pharmacy, Shahid Beheshti University of Medical Sciences, Tehran, Iran

**Keywords:** Silver, Silica, Nanoparticle, Skin lesions, Inflammatory cytokine, Genetics, Immunology, Molecular biology, Environmental sciences, Environmental social sciences, Diseases, Health care, Medical research, Molecular medicine, Materials science, Nanoscience and technology

## Abstract

Occupational health must be strictly considered in industries particularly in nanoparticle factories where workers were exposed to different types of chemicals. We measured the serum levels of inflammatory cytokines in workers who developed skin lesions after exposure to silver and silica nanoparticles. Using a questionnaire in this cross-sectional study, we identified 110 workers in nanoparticle industries who were exposed to silver and silica nanoparticles. We also included 40 healthy subjects as controls from the administrative department of the same factories who were not exposed to nanoparticles. Peripheral blood samples used to measure the mRNA levels of inflammatory cytokines by qRT-PCR. In comparison with the control group, the workers who developed skin lesions had significantly higher levels of interleukin IL4, IL6, IL8, and TNF-α, particularly after two or three decades of exposure to silver and silica nanoparticles. Participants who were exposed to silver had higher levels of IL6 and IL8 compared with those who were exposed to silica. Necessary measures must be considered to protect workers in nanoparticle industries against the potential toxic effects of these compounds. Our network pharmacology study suggests corresponding biochemical pathways for these disorders.

## Introduction

As part of preventive medicine, occupational health has increasingly received attention during recent decades, which can substantially decrease the cost of treatment and long-term mortality and morbidity^1^. Occupational health can guarantee higher rates of productivity and improve ergonomics in workplaces^1,2^. With the increasing use of nanoparticles, particularly silver and silica nanoparticles, a higher incidence of human and environmental exposure can be expected, particularly in nanoparticles industries^3^. Due to the potential risk of detrimental health outcomes, preventive measures should always be taken into account^3^.

Silver nanoparticles can induce hepatic, respiratory, vascular, nervous, and dermatologic adverse effects^3,4^. Similarly, previous studies have attributed several serious complications, such as myositis, lung fibrosis, and cutaneous fibrosis, to silica nanoparticles^5^. A review of preclinical studies has shown that silica nanoparticles are potentially toxic for several systems such as the nervous system, digestive system, urinary system, and respiratory system^6^.

Silica nanoparticles can interfere with various cellular processes such as oxidative stress, autophagy, endoplasmic reticulum stress, apoptosis, and cellular energy metabolism^6^. Silica exposure was shown to impair the immune response, thereby increasing the odds of autoimmunity^6^. Similarly, it has been unveiled that accumulation of silver nanoparticles can lead to reactive oxygen species (ROS) accumulation, lipid peroxidation, mitochondrial dysfunction, DNA damage, and apoptosis^7^.

There is usually an association between dermatologic symptoms and the systemic effects of different chemicals^8^. Besides, nanoparticles can be absorbed through the skin, gastrointestinal tract, and respiratory tract in the workplace; therefore, apart from their possible dermatologic adverse effects, systemic toxicities are also possible^9^. The dermatologic manifestations of exposure to nanoparticles may be linked with immune system parameters which might get worse over time. Environmental factors exacerbate over time, causing harm to the skin's protective barrier. This damage triggers the activation of the adaptive immune system, resulting in lymphocyte differentiation. Subsequently, the activation of Th1 leads to heightened secretion of TNF, IL8, and a chronic response. Stimulation of Th2 results in increased secretion of IL4, B lymphocytes, eosinophils, and an acute response. This damage triggers the activation of the innate immune system, affecting pattern recognition receptors (PRR) and leading to the activation of pathogen-associated molecular patterns (PRR) and R753Q Toll-like receptors (TLR2), associated with an increase in IL6^10^. In this study, our research team designed a study to investigate the serum levels of inflammatory cytokines in workers with different durations of exposure to silver and silica nanoparticles who have developed skin lesions in the form of contact dermatitis to understand this process.

## Materials and methods

### Participants

Workers who had chronic exposure to silver and silica nanoparticles in nanoparticles industries were first screened using a questionnaire. Informed consent was obtained from all subjects and/or their legal guardians. The study protocols were conducted according to the principles of the Declaration of Helsinki (version 2013) and were approved by the Ethics Committee of Shahid Beheshti University of Medical Sciences (IR.SBMU.retech.rec.1399.721). We selected participants who were employed in silver and silica nanoparticles industries. The experienced doctor of the project visited the workers; all of them had skin health history and without any signs of genetic disease related to the skin with no positive family history. Active (first-hand) smokers and passive (second-hand) smokers, individuals with high blood pressure and food allergies were excluded from this study. In total, 110 workers who had chronic exposure (Exposure for more than one year was considered as chronic exposure) to silver and silica nanoparticles were included in this cross-sectional analytical study. Workers were divided into three groups based on the duration of exposure and the type of nanoparticles they were exposed to. We also included 40 employees from the same industries who were the personnel of the administrative department and had neither exposure to silver nor silica nanoparticles as the control group. Demographic characteristics such as age and gender, allergy history, past medical history, and dermatologic symptoms were collected separately for each group (case and control, Supplementary Table 1a & b). Specifically, we assessed the presence of flaky skin, skin induration and roughness, hair loss, redness, chap, rash, sweating, skin irritation, skin lightening, and mole. One mL of peripheral blood sample was collected from each participant for measuring the serum levels of inflammatory cytokines.

### Inclusion criteria

Workers with no prior history of pre-existing skin abnormalities or genetic skin diseases, or a family history of skin disorders.

### RT-PCR analysis

The key cytokines crucial for the development of inflammatory skin issues were chosen for investigation in this study. PCR was used to measure the gene expression of IL4, IL6, IL8, and TNF-α RNX plus solution (CinnaGen Co., Iran) was used to extract RNA from blood samples. Briefly, 200 μL of blood sample was added to 800 μL of RNX plus solution, shaken for 5–10 s, and incubated at room temperature for 5 min. Then, it was incubated with 200 μL of chloroform, shaken for 15–30 s, and kept at − 20 °C for 5 min. Then, the sample was centrifuged (12,000*g*) at 4 °C for 15 min. The supernatant (800 μL) was separated and mixed with the same volume of isopropanol. Then, the solution was kept at − 20 °C for 1–2 h and centrifuged (12,000*g*) at 4 °C for 20 min. The supernatant was substituted with 500 μL of 70% ethanol and the solution was centrifuged (7500*g*) at 4 °C for 3 min. To ensure RNA concentration, we used nanodrop spectrophotometer to read RNA-specific bands (8S, 18S, and 28S) on agarose 1% gel.

We used Aryatos cDNA kit for cDNA synthesis. Briefly, 5 μL of RNA was mixed with 10 μL of Buffer-Mix and 2 μL of Enzym Mix. DEPC-treated water was added to reach the final volume of 20 μL. The solution was heated at 25 ֯C for 10 min, then at 60 °C for 47 min, and finally at 85 °C for 5 min. The cDNA was used for PCR or stored at − 80 °C. Primer was designed by Primer 3 software (Table 1).

**Table 1:** The used primers for PCR.

Analyte	Primer
IL4	F: CTTTGCTGCCTCCAAGAACAC R: GCGAGTGTCCTTCTCATGGT
IL6	F: GGTACATCCTCGACGGCATC R: CACCAGGCAAGTCTCCTCATT
IL8	F: CGCCAACACAGAAATTATTGTAAAG R: AACTTCTCCACAACCCTCTG
TNF Alpha	F: CCAGGGACCTCTCTCTAATCA R: TCAGCTTGAGGGTTTGCTAC
GAPDH	F:CGACAGTCAGCCGCATCTTC R:CCCAATACGACCAAATCCGTTGA

The following primers were used for PCR:

We used master mix PCR for performing PCR. First, the samples were kept at 95 °C for 2 min and then they were introduced to the device based on the following protocol:

$$\left\{ {\begin{array}{*{20}l} {95\;^{^\circ } {\text{C}}\;30\;{\text{s}}} \hfill \\ {60\;^{^\circ } {\text{C}}\;30\;{\text{s}}} \hfill \\ {72\;^{^\circ } {\text{C}}\;30\;{\text{s}}} \hfill \\ \cdots \hfill \\ {72\;^{^\circ } {\text{C}}\;5\;{\text{min}}} \hfill \\ \end{array} } \right\} \times 35$$

The volume of each component for PCR were as follows: Master Mix (10 µL), primer forward (PF) (0.5 µL), primer reverse (PR) (0.5 µL), cDNA (2 µL), and DEPC water (7 µL). Dnase-free Water was used instead of cDNA for negative control samples. After preparing agarose gel and electrophoresis buffer, 9 μL of samples and 4 μL of 1 kb marker were added to each well, and we ran electrophoresis (92 V, 20–25 min). Using gel doc, the gels were imaged under ultraviolet light.

### Real-time PCR

The volume of each component for real-time PCR were as follows: Master Mix (10 µL), primer forward (PF) (0.5 µL), primer reverse (PR) (0.5 µL), cDNA (2 µL), and DEPC water (7 µL).

### C Reactive Protein (CRP test)

We used ENISON CRP kit for CRP test. Usually, the slide method is used qualitatively at first. For this purpose, a drop of the individual's serum after deactivation (placed at 56° for 30 min) is placed on a slide with a black background (next to the positive and negative controls) and then a drop of antibody solution attached to latex, which is A commercial form is prepared, they add. After mixing with the applicator, rotate the slide on the rotator for 3 min and the result is read in front of the reading light. Usually, in this case, the results are reported as Neg (−), Pos (+), Pos (+2), Pos (+3), Pos (4).

### Network Pharmacology Study

The network Pharmacology was used to investigate the pathways that lead to the occurrence of inflammation or autoimmune responses.

### Statistical analysis

GraphPad Prism version 8 was used for analyzing data. All bar charts are presented as mean ± SD. We applied student’s t-test to compare non-exposed participants (control group) with those who were exposed to nanoparticles and developed skin lesions based on their length of exposure. P < 0.05 was deemed statically significant. Student’s t-test analysis was performed on all silica and silver samples together. In addition, Co-variance analysis (ANCOVA) was conducted to assess the significance of age, gender, education, and other factors in skin disorders, but none of them were found to be significant (P > 0.05) co-variance in this study.

Finally, the Fisher's exact test was applied to measure the effect of exposure length (decades) and development of symptoms.

## Results

### Chronic exposure to silver and silica nanoparticles markedly increased the genes expression of IL8, TNF-α, IL4 and IL6 in workers who developed skin lesions

**Figure 1:**
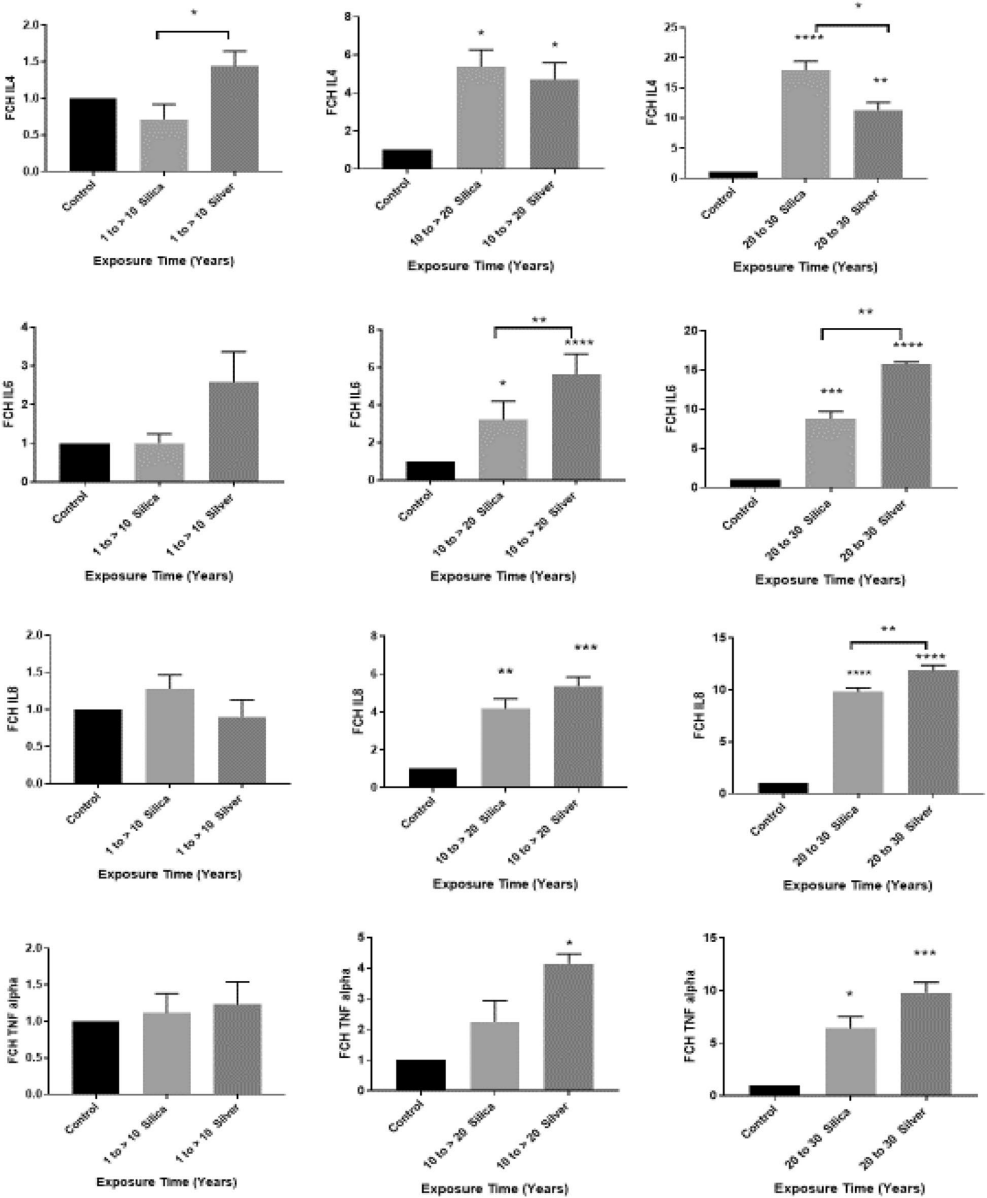
The genes expression of IL8, TNF-α, IL4 and IL6 in workers who developed skin lesions after prolonged exposure to silver and silica nanoparticles. Participants were divided based on the length of exposure. The genes expression of IL8, TNF-α, IL4 and IL6 were compared between the healthy control group who did not have exposure to nanoparticles and workers who developed skin lesions after prolonged exposure to silver and silica nanoparticles. Student’s t-test was used to compare the control group with each one of the nanoparticle groups or the nanoparticle groups with each other. *P < 0.05, **P < 0.01, ***P < 0.001, ****P < 0.0001. Data are presented as mean ± SD.

**Figure 2:**
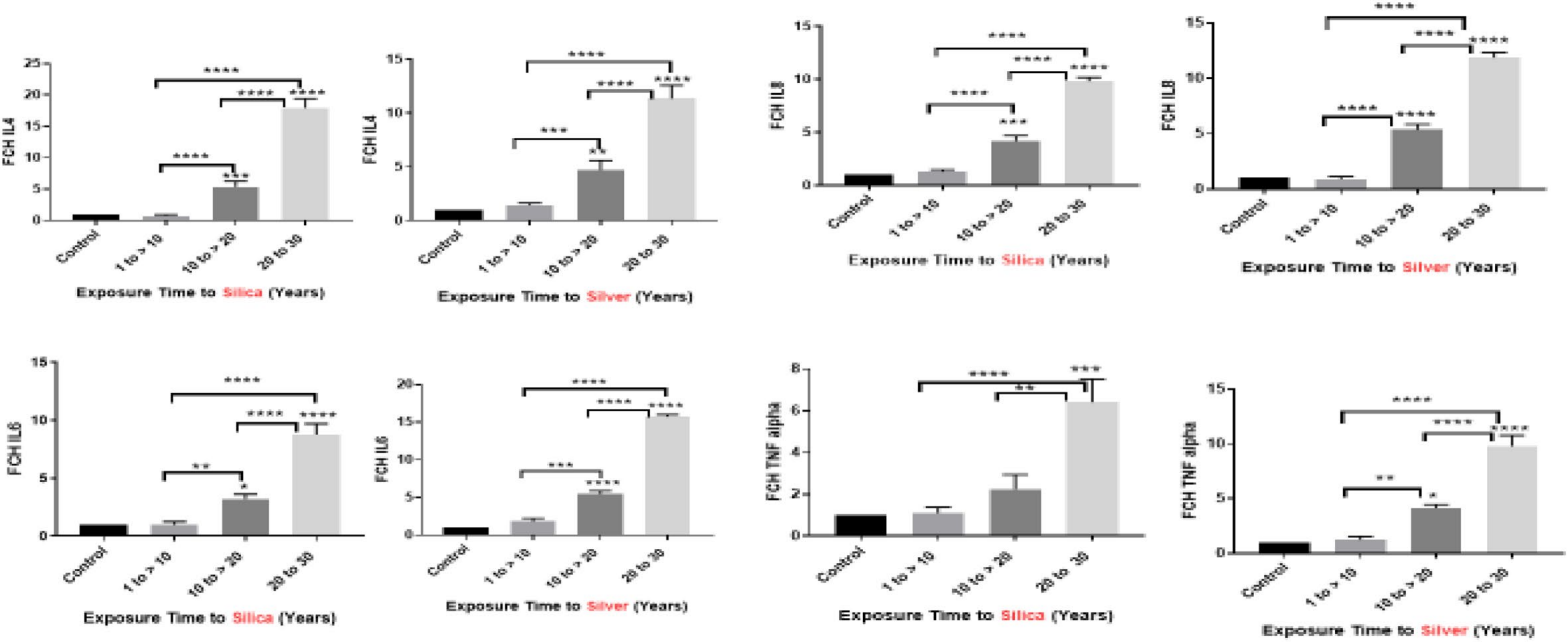
The genes expression of IL8, TNF-α, IL4 and IL6 based on decades of exposure to silver and silica nanoparticles. The genes expression of IL8, TNF-α, IL4 and IL6 were compared between groups with different decades of exposure. Student’s t-test was used to compare the control group with each one of the nanoparticle groups or the nanoparticle groups with each other. *P < 0.05, **P < 0.01, ***P < 0.001, ****P < 0.0001. Data are presented as mean ± SD.

The PCR results presented as fold change (FCH) have shown that the mRNA levels of IL8, TNF-α, IL4, and IL6 were markedly upregulated in participants who developed skin lesions after prolonged exposure to silver and silica nanoparticles in their workplaces compared to healthy controls who did not have exposure to nanoparticles. Exposure to silver nanoparticles significantly elevated the genes expression of IL8, TNF-α, IL4 and IL6 after two (P < 0.001 for IL8, P < 0.05 for TNF-α, P < 0.0001 for IL6 and P < 0.05 for IL4) or three (P < 0.0001 for IL8, P < 0.0001 for TNF-α, P < 0.01 for IL4 and P < 0.0001 for IL6) decades of exposure. Similarly, exposure to silica nanoparticles significantly elevated the genes expression of IL8 after two (P < 0.01) and three (P < 0.0001) decades of exposure. Exposure to silica nanoparticles led to a significant increase in TNF-α levels (P < 0.05) only after three decades of exposure. Moreover, IL8 levels were significantly (P < 0.01) higher in those who were exposed to silver nanoparticles compared with those who were exposed to silica nanoparticles. Similarly, exposure to silica nanoparticles significantly elevated the genes expression of IL4 and IL6 after two (P < 0.05) or three (P < 0.0001 for IL4 and P < 0.001 for IL6) decades of exposure. IL6 levels were significantly (P < 0.01) higher in those who were exposed to silver nanoparticles compared with those who were exposed to silica nanoparticles. In the first decade of exposure, IL4 levels were significantly (P < 0.05) higher in those who received silver nanoparticles, while IL4 levels were markedly (P < 0.05) higher in those who received silica nanoparticles after three decades of exposure (Fig. 1). Workers who were exposed to silver and silica nanoparticles had several folds higher mRNA levels of IL8, TNF-α, IL4 and IL6 after three decades of exposure, and their genes expression continuously increased with the decades of exposure (Fig. 2).

### CRP test

C-Reactive Protein is used as a severity criterion, and all samples showed inflammation, with two positives (++) confirming the real-time PCR result.

### Network Pharmacology Survey

**Figure 3:**
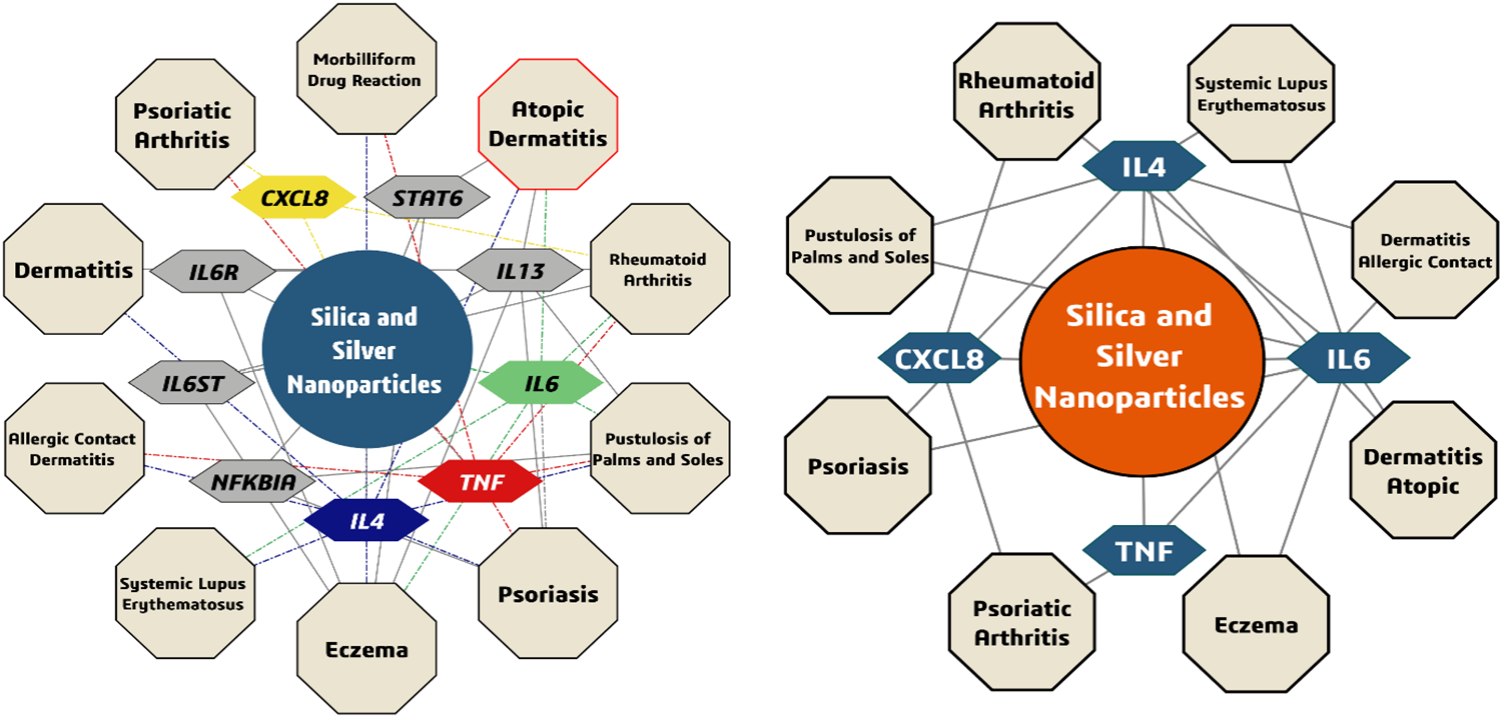
The graph of Network Pharmacology Survey and also diseases related interpretation of Network Pharmacology.

The graph of Network Pharmacology Survey shows that the effect of silica and silver nanoparticles on the expression of various genes such as IL4, IL6, IL8 and TNFα leads to the occurrence of various skin diseases such as atopic dermatitis. Which is in line with our survey on human samples (Fig. 3).

In Fig. 3, the rash was not significantly associated with increased IL4 levels, while dry skin is significantly linked to elevated IL4 levels in the Diseases-related interpretation of Network Pharmacology.

Silica and Silver associated genes were curated through a comprehensive literature review of recent articles. Subsequently, we identified four specific targets (IL4, IL6, IL8, and TNF) related to Silica and Silver. We used them in a search within the DisGeNET online database (https://www.disgenet.org/; accessed on October 11, 2023) with a filtering criterion of a "GDA score ≥ 0.4" to uncover potential disease associations. All skin system-related diseases were manually selected, and their network pharmacology graph was constructed using Cytoscape 10.1^11^.

### Statistical survey

#### Demographic comparison

Demographic factors such as age and sex in control and case groups with different times of exposure to nanoparticles were not statistically different.

#### The association between the length of exposure (decades) and development of symptom

Fisher’s exact test revealed that there was no significant association between the length of exposure and development of blood pressure, cough, sore throat, dyspnea, wheeze, hair loss, sweating, skin irritation, and skin lightening, whereas the length of exposure was significantly associated with sneeze (P < 0.001), flaky skin (P < 0.002), skin roughness (P < 0.01), chap (P < 0.03), mole (P < 0.05), rash (P < 0.05), and skin redness (P < 0.05).

#### Student′s t-test analysis for measuring the association between symptoms and cytokine levels

After performing student′s t-test analysis, we found that gender, skin redness, rash, hair loss, skin lightening, skin irritation, and sweating did not significantly correlate with IL4 level, while nanoparticle exposure (P < 0.001), flaky skin (P < 0.003), skin roughness (P < 0.007), chap (P < 0.002), and mole (P < 0.048) significantly correlated with IL4 levels in student′s t-test. Similarly, gender, skin redness, chap, hair loss, skin lightening, skin irritation and sweating did not significantly correlate with IL6 level, whereas nanoparticle exposure (P < 0.001), flaky skin (P < 0.001), skin roughness (P < 0.003), rash (P < 0.014), mole (P < 0.005) markedly correlated with IL6 level expression in student′s t-test. Gender, skin redness, hair loss, skin lightening, skin irritation and sweating did not significantly correlate with the IL8 expression in student′s t-test, whereas nanoparticle exposure (P < 0.001), flaky skin (P < 0.001), skin roughness (P < 0.003), chap (P < 0.004), rash (P < 0.014), and mole (P < 0.003) markedly upregulated it. Likewise, gender, skin redness, hair loss, skin lightening, skin irritation and sweating did not significantly correlate with the TNF-α levels, whereas nanoparticle exposure (P < 0.001), flaky skin (P < 0.001), skin roughness (P < 0.002), chap (P < 0.018), rash (P < 0.036), and mole (P < 0.11) are significantly correlated with the TNF-α expression. In all calculations, comparisons were made in respect to the control group in this study.

## Discussion

In this study, we examined the skin impacts of long-term exposure to certain nano-materials. Decades of employment in these industries were chosen as the relevant comparison periods due to insufficient yearly samples and the slow development of skin disorders. We found that participants with skin lesions in form of contact dermatitis caused by chronic exposure to silver and silica nanoparticles had higher genes expression of inflammatory cytokines, including IL4, IL6, IL8, and TNF-α compared to control individuals. Furthermore, we have also showed that a longer duration of exposure was associated with higher levels of these cytokines and a more severe systemic inflammatory response. C Reactive Protein was measured after the exposure in all workers exposed to nanoparticles, which showed systemic inflammation. The factories were mostly working on nano-material products, and there were no indications of pre-existing well-known toxic agents. In any case, Isfahan is an industrial city, and low plasma cytokine levels may be associated with air pollution. Nevertheless, the control group is included to address issues like this. Both the control and subjects reside in the same area, just a few meters apart, and the primary distinction between these groups is the close exposure to nanoparticles by the subjects. Clearly, those who developed symptoms had higher levels of inflammatory cytokines compared to those who did not develop the symptoms. Exposure to silver nanoparticles can result in immune dysregulation, characterized by a lower abundance of tolerant T cells and a higher abundance of inflammatory cytokines such as IL6^12^. Consistently, exposure to silver nanoparticles was shown to promote the gene expression of pro-inflammatory cytokines such as IL-1, IL-6, and TNF-α in human monocytes^13^. Consistently, we found that exposed participants had higher levels of IL4, IL6, IL8, and TNF-a, and length of exposure was significantly associated with the development of some of the symptoms.

Interestingly, it has been observed that silver nanoparticles can prime mast cells and enhances their degranulation and activation in response to allergens^14^. It was shown that silver nanoparticles increase ROS production and leukotriene B4 and IL6 release after exposure to an allergic antigen^14^. Similarly, Kang et al*.* indicated that silver nanoparticles exacerbate the dermatologic manifestation of atopic dermatitis in mice through mast cell over activation^15^. Mechanistically, silver nanoparticles can activate nuclear factor кB (NF-кB), which is a master regulator of an inflammatory response and a major transcription factor for numerous inflammatory cytokines^16^.

Samberg et al*.* reported that unwashed silver nanoparticles can dose-dependently decrease the viability of cultured human epidermal keratinocytes^17^. They also found that unwashed silver nanoparticles markedly increased the release of IL1β, IL6, IL8, and TNF-α^17^. Besides, topical exposure to silver nanoparticles for 14 days led to focal inflammation and localization of silver nanoparticles on the surface and in the upper stratum corneum layers of skin in a porcine model^17^. Silver nanoparticles can locally penetrate as deep as the reticular dermis, which may warrant higher levels of inflammatory cytokines in subjects of our study who experienced skin lesions^18^. Furthermore, silver nanoparticles can be absorbed through other routes such as respiration, which increases their systemic effects^19^.

Similar to silver nanoparticles, small-sized silica nanoparticles can effectively penetrate the skin barrier^20^. Consistently, Hirai et al*.* indicated that small-sized amorphous silica nanoparticles can penetrate the skin barrier, induce T helper 2 response, promote inflammatory cytokine release, and aggravate dermatologic manifestations in a mice model of atopic dermatitis^21^. Treatment with silica nanoparticles increased the circulatory levels of IL-1β and TNF-α in mice^22^. Furthermore, it was observed that silica nanoparticles potentiated the ability of murine macrophages to produce inflammatory cytokines^22^. Even, a single dose of intraperitoneally administered (0.25 mg/kg) silica nanoparticles (50 nm) in a mice model led to the overproduction of ROS and inflammatory cytokines in several organs such as the heart, liver, brain, and lung^23^.

Interestingly, according to student′s t-test, we found that some skin conditions, such as flaky skin (P < 0.001), skin roughness (P < 0.003), chap (P < 0.004), rash (P < 0.014), and mole (P < 0.005), significantly affected inflammatory cytokine expression, whereas other skin conditions, such as gender, skin redness, rash, hair loss, skin lightening, skin irritation, and sweating, could not markedly alter the expression levels of IL4, IL6, IL8, and TNF-α (P > 0.05). This observation may be warranted by different severity of the inflammatory response in different skin conditions. It also implies that anti-inflammatory agents may be more effective in some of these skin conditions.

This study had some limitations. First, due to the unwillingness of factory managers to cooperate and maintain confidentiality conditions regarding their materials details and working conditions, we do not have information about the size of nanoparticles and their nature of nanoparticles. Second, due to limitation in contact with factory workers, sample collection, and staff access symptoms were mainly self-reported by participants instead of being objectively assessed by a physician.

Network pharmacology indicates that the inflammatory genes we studied are the most relevant pathways for the mechanisms causing allergy-like autoimmune disorders during prolonged exposure to nanomaterial, as depicted in Fig. 3.

## Conclusion

The project focused on workers without pre-existing skin abnormalities, genetic skin diseases, or a family history of skin disorders. Although it was not a criterion of selection for us, many of them claimed to have developed skin lesions gradually during their employment. Workers who had prolonged exposure to silver and silica nanoparticles and developed skin lesions had higher genes expression of inflammatory cytokines compared with normal subjects who had no exposure to silver and silica nanoparticles. The genes expression of inflammatory cytokines continuously increased with the length of exposure, suggesting the role of chronic exposure to silver and silica nanoparticles in immune dysregulation. Necessary measures must be considered to protect workers in nanoparticle industries against the detrimental effects of these nanoparticles. A network pharmacology study has presented suggestions for corresponding biochemical pathways for these disorders.

### Supplementary Information

Supplementary Information.

## Data Availability

The datasets used and/or analyzed during the current study are available from the corresponding author on reasonable request. Please contact the corresponding author for the data requests.
