# Supplementary material for: High inflammatory cytokines gene expression can be detected in workers with prolonged exposure to silver and silica nanoparticles in industries
Source: Sci Rep. 2024 Mar 7;14:5667. doi: 10.1038/s41598-024-56027-z (PMC11319723; doi:10.1038/s41598-024-56027-z)
Supplement: Supplementary file 1 — Supplementary Information. [file 41598_2024_56027_MOESM1_ESM.pdf]

Supplementary

Questioner:

The questioner was filled anonymously for each of participating persons in this study and Demographic characteristics

In the name of god

This questionnaire contains 24 questions which has two components: personal information and employee health information. Please read each of these sentences carefully and indicate your opinion by marking yes or no. It should be noted that all personnel information is confidential without mentioning names and surnames.

1/ age:		
2/ Sex:	Male:	Female:
3/ Education:		
4/ Workplace:		
5/ Daily working hours:		
6/ Work experience:		
7/ In which unit are you working?		
8/ How many years have you been working in this unit?		
9/ Do you familiar with Nano products?	YES:	NO:
9/A Do you use devices containing Nano?	YES:	NO:
9/B Which nanomaterials are you familiar with?		
10/ The chemical composition of the Nano material that you are in contact with?		
11/ What is the physical state of the Nano material that you are in contact with?		
12/ Do you have a disease?<br>blood pressure :------- fasting sugar:---- body temperature: ------ berating rate:-----	YES:	NO:
13/ Do you smoke?	YES:	NO:
14/ Do you have a history of allergies?	YES:	NO:
15/ Do you have a history of hereditary disease?	YES:	NO:
16/ Do you have a history of hereditary disease in your relatives or close family?	YES:	NO:
17/ Do you take medicine?	YES:	NO:
18/ Do you take vitamin pills?	YES:	NO:
19/ Have you noticed a change in your health in the last two years?	YES:	NO:
20/ Which of the **symptoms of skin disease** do you have?		
20/A Since I have been working in this job, I have **dry skin**	YES:	NO:
20/B Since I have been working in this job, I have **flaky skin**	YES:	NO:
20/C Since I have been working in this job, I have **reddened skin**	YES:	NO:
20/D Sine I have been working in this job, I get **skin irritation**	YES:	NO:
20/E Sine I have been working in this job, **my skin has become lighter**	YES:	NO:
20/F Since I have been working in this job, **my skin has become softer**	YES:	NO:
20/G Since I have been working in this job, **my skin has become rougher**	YES:	NO:
20/H Since I have been working in this job, I get **skin sores**	YES:	NO:
20/I Since I have been working in this job, I get **skin moles**	YES:	NO:
20/J Since I have been working in this job, I get **acne**	YES:	NO:
20/K Since I have been working in this job, I have **increased hair loss**	YES:	NO:
20/L Since I have been working in this job, I have **reduced hair loss**	YES:	NO:
20/M Since I have been working in this job, I **sweat more**	YES:	NO:
20/N Since I have been working in this job, I **sweat less**	YES:	NO:

21/ Do you have a medical screening program including initial examinations to prevent occupational diseases?	YES:	NO:
22/ Has a special disease been diagnosed in the initial examinations?	YES:	NO:
23/ Do you have a medical screening program including periodical examinations to prevent occupational diseases?	YES:	NO:
24/ Has a specific disease been diagnosed in periodic examinations?	YES:	NO:

After the completion of the project and obtaining the final data, the test result will be provided to the factory manager. The research team thanks all personnel for their help in conducting this research.

Supplementary table 1 a:

1 a) Biological parameters obtained from workers (cases).

**Case:**	**110**
**Age (yrs.):**	**25 – 60 Y**
**Sex (M/F) Case :**	**Men : 85 Female: 25**
**Smoker:**	**never-smoker**
**Region:**	**Isfahan**
**History of hereditary disease in close relatives:**<br>**Blood pressure :**	 **1**
**Skin disease after exposure:**<br>**Flaky skin:**<br>**Skin roughness:**<br>**Hair loss:**<br>**Skin redness:**<br>**Chap:**<br>**Rash:**<br>**Sweating:**<br>**Skin irritation:**<br>**Skin lightening:**	 **10**<br>**8**<br>**12**<br>**8**<br>**6**<br>**4**<br>**4**<br>**5**<br>**5**
**Genetic skin disease:**	**0**
**Average blood pressure:**	**13.50 / 70**
**Average fasting sugar:**	**80 – 140 mg**
**Average body temperature:**	**37 C**
**Average breathing rate :**	**15 – 22 times/ minutes**

Supplementary table 1 b:

1 b) Biological parameters obtained from control.

**Control:**	**40**
**Age (yrs.):**	**25 – 60 Y**
**Sex (M/F) Control:**	**Men: 25 Female: 15**
**Smoker:**	**never-smoker**
**Region:**	**Isfahan**
**History of hereditary disease in close relatives:**<br>**Blood pressure :**	 **4**
**Skin disease after exposure:**<br>**Flaky skin:**<br>**Skin roughness:**<br>**Hair loss:**<br>**Skin redness:**<br>**Chap:**<br>**Rash:**<br>**Sweating:**	 **0**<br>**0**<br>**2**<br>**0**<br>**0**<br>**0**<br>**2**
**Skin irritation: Skin lightening:**	**0**<br>**0**
**Genetic skin disease:**	**0**
**Average blood pressure:**	**12.50 / 80**
**Average fasting sugar:**	**80 – 125 mg**
**Average body temperature:**	**37 C**
**Average breathing rate :**	**13 – 21 times/ minutes**
